# Surgical-related Morphological Characteristics of Sphenoid Sinuses: A Comprehensive CT-Based Analysis

**DOI:** 10.4317/jced.62099

**Published:** 2024-12-01

**Authors:** Diego Santiago de Mendonça, Nadya Imani Newman, João Edson Ribeiro Leite, Esther Carneiro Ribeiro, Marcela Lima Gurgel, Cauby Maia Chaves Júnior, Lucia Helena Soares Cevidanes, Lúcio Mitsuo Kurita, Paulo Goberlânio de Barros Silva, Fábio Wildson Gurgel Costa

**Affiliations:** 1 Postgraduate student, Universidade Federal do Ceará, Fortaleza, Ceará, Brazil; 2 Postgraduate student, no affiliation, Universidade Federal do Ceará, Fortaleza, Ceará, Brazil; 3 PhD, Universidade Federal do Ceará, Fortaleza, Ceará, Brazil; 4 PhD, University of Michigan, Ann Arbor, United States

## Abstract

**Background:**

This study aims to assess the relationships between sphenoid sinus (SS) types, septation, lobulation, symmetry, septal deviation, and the variations in SS pneumatization regarding the surrounding neurovascular structures using Computed tomography (CT) images. Sexes and age groups were investigated.

**Material and Methods:**

We retrospectively evaluated head CT-scans of 320 patients (age range 18–49 years); mean of 43.13 ± 17.39 to evaluate the morphological characteristics of the SS (Symmetry, Pneumatization, Extension, Septation, Lobulation, Internal Carotid, and Optic Nerve). Analyses were performed using SPSS version 20.0 (IBM Corporation, Armonk, NY, USA), with a 95% confidence level.

**Results:**

Our findings revealed an incidence of anatomographical variations in terms of pneumatization that varied between 0.3% (conchal)–60% (postsellar). These variants include 72.5% subdorsal extension, 92.2% lobular extension. Septations either presented as complete septa (90.9%) which extend from the anterior to the posterior wall dividing the sinus into different antra or as incomplete accessory septa. We also demonstrated anatomographic variants in terms of Internal Carotid (ICA), and Optic Nerve (ON). As sphenoid sinuses pneumatize more, the frequency of optic nerve (ON) and internal carotid artery (ICA) protrusion and wall dehiscence into the sinus increases.

**Conclusions:**

Results show that anatomic variations and pneumatization of the paranasal sinuses holds significant importance in diagnosing and understanding sinus pathologies The findings underscore a potential correlation between the anatomical variants of the SS and the presence of population group variability.

** Key words:**Sphenoid sinus, CT scan, •Sellar type, Internal carotid artery, Optic nerve.

## Introduction

The sphenoid sinuses (SS) are two air filled spaces located at the base of the skull, within the sphenoid bone. Although they develop primarily after puberty, they appear as small cavities at birth. In early childhood, they extend posteriorly into the presellar region, reaching their maximum size in adolescence and expanding to the inferior-posterior region of the sella turcica (1,2).

SS exhibit diverse anatomical characteristics influenced by their pneumatization, position relative to the sella turcica, presence of septa, lobes, and symmetry. Based on their position in relation to the anterior and posterior walls of the sella turcica in the sagittal plane, and the extent of pneumatization in the clivus, they can be classified into conchal, presellar, sellar, and postsellar types. Additionally, in terms of their position in relation to the vidian canal, posterior wall, and floor of the sella turcica, the extent of pneumatization in the clivus is categorized as subdorsal, dorsal, occipital, and combined (dorsal and occipital) types. Furthermore, the SS may exhibit symmetry or asymmetry and can be divided into lobes, presenting as bi or multilobulated (3).

Numerous crucial structures, including the pituitary gland, optic nerve (ON), and internal carotid artery (ICA), are located close to the SS. Because the ON and ICA may touch the sinus or protrude within it, knowledge and assessment of these anatomical variations is pertinent to endoscopic surgical planning to avoid possible complications (4).

Computed tomography (CT) with multiplanar reconstructions is used to not only assess size, shape, sex, craniofacial features, and volume of the SS but also perform linear and angular measurements (2,5). The potential value of CT scans for determining the probable cause of death, estimating age, or visualizing characteristics that may allow personal identification has been reported in the literature in a forensic context (6), CT represents a precise imaging technique with high cost-effectiveness compared to two-dimensional radiographic modalities (7,8).

Given the proximity with several neurovascular structures, the anatomic evaluation of the SS is crucial for successful transsphenoidal surgical access (9). However, research on the role of sex and age in the SS anatomy, particularly in the context of the Brazilian population, remains scarce. Thus, this study aims to assess the anatomic characteristics of the SS, including (a) extension, (b) septal deviation, (c) lobulation, (d) pneumatization, (e) symmetry, and (f) the presence of neurovascular structures such as the internal carotid artery and optic nerve in CT scans. Additionally, it evaluates the role of sex-specific and age stratification data on these SS anatomic features.

## Material and Methods

-Study design and ethics statement

This multicentred cross-sectional investigation using CT scans was performed after receiving approval from the Ethics and Research Committee of the Federal University of Ceará (number 61257622.0.0000.5054) in accordance with the principles of the Declaration of Helsinki. It was conducted according to the Strengthening the Reporting of Observational Studies in Epidemiology (STROBE) Statement (10).

-Sample

The study included 320 tomographies (of 146 men and 174 women) from imaging centers located in northeastern Brazil (Bahia, Pará, and Ceará). One investigator initially analyzed the hospital image database until the necessary sample was obtained, as it included all the CT scans required for different clinical purposes (e.g., cranial trauma and maxillofacial injuries assessment). The inclusion criteria were imaging exams from individuals clearly displaying at least one of the following: duplicated exams, images revealing signs of pathology or fractures, indications of facial growth disorders, or craniofacial syndromes. Exclusion criteria included motion artifacts and low-quality diagnostic images that could impede the assessment of sinus-related structures.

Regarding the sample size estimation, we considered a study by Akgül *et al*. (2016) (11) which revealed a relationship between septal deviation and SS types, septation (lobulation), and symmetry. Student’s t-test estimated 320 tomographic images to obtain a representative sample (90% of power and assuming a 95% confidence interval).

-Variables

Tomographic data were obtained using a single scanner (Somatom Emotion 6, Siemens, Forchheim, Medical Solutions, Germany) under the following acquisition protocol: 1mm of Table increment, 130 kVp, milliamperage ranging from 80 to 120 mA, cross-section image thickness up to 2.0 mm, 180 mm FOV, and 0.6 seconds of rotation time. The same computer (Dell Inc., model G3 3590, Intel® Core ™ processor i5-9300H CPU @ 2.40GHz, 2400 Mhz, 4 colors, 8 logic processors - LED HD backlight screen) was used to perform all analyses, and Digital Imaging and Communications in Medicine (DICOM) files were imported to the free software TK-SNAP version 3.8.034. The morphological characteristics of the SS were evaluated based on the classifications presented in  Table 1.

**Figure 1:**
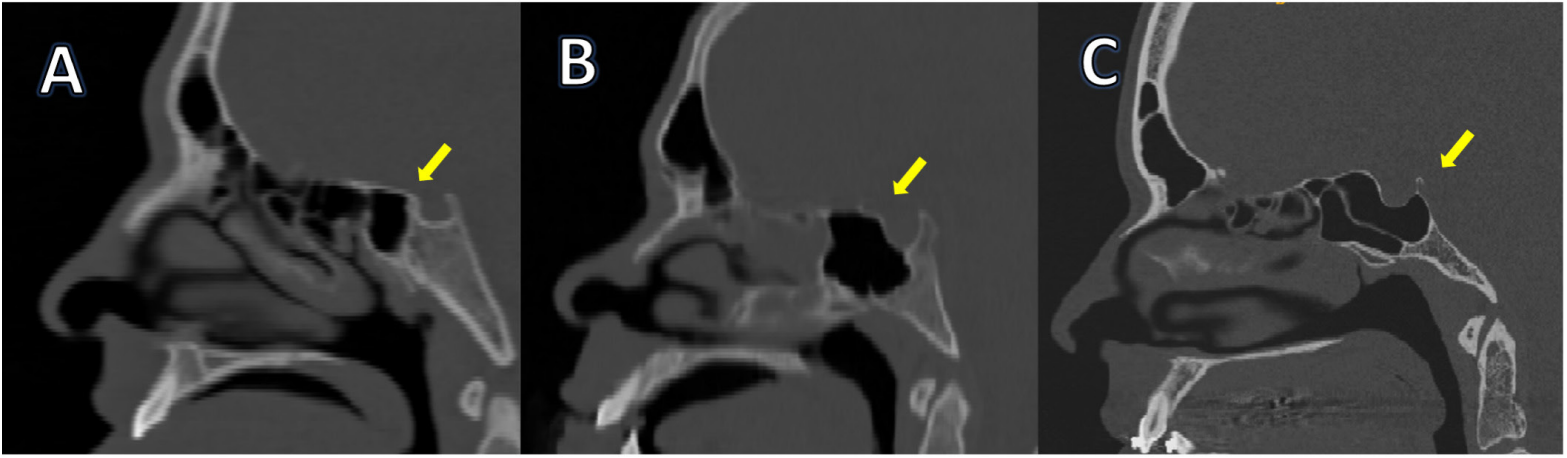
Sagittal paranasal computerized tomography. A) Presellar; B) Sellar; and C) Postsellar types of SS was demonstrated.

**Figure 2:**
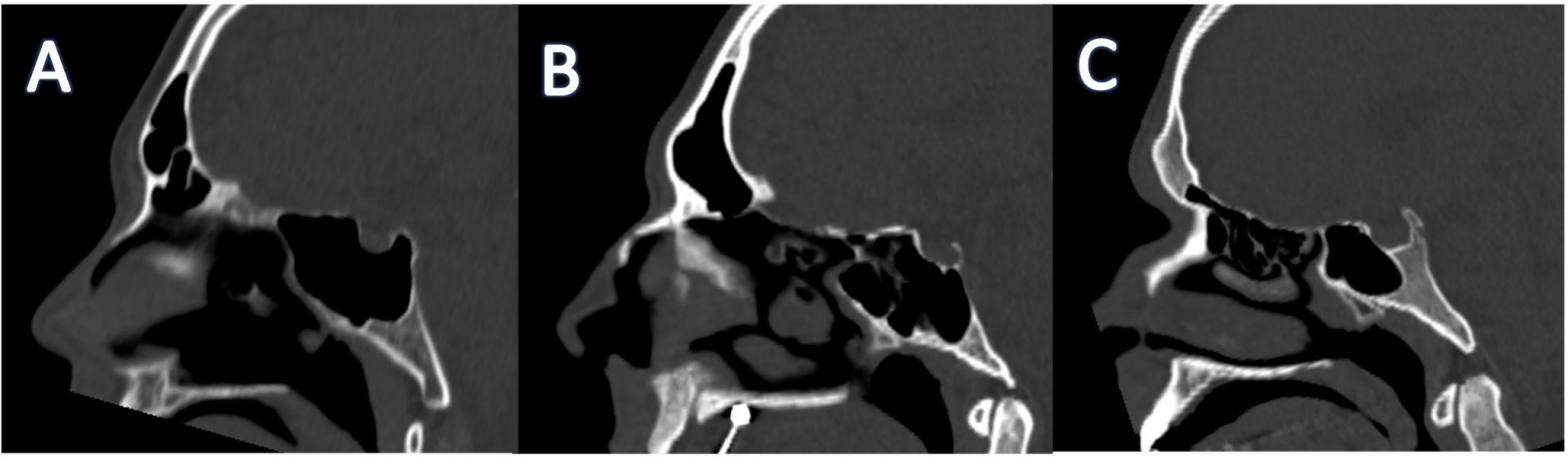
Different types of sphenoid types of clival extension: A) Dorsal; B) Subdorsal, C) Occipital.

**Figure 3:**
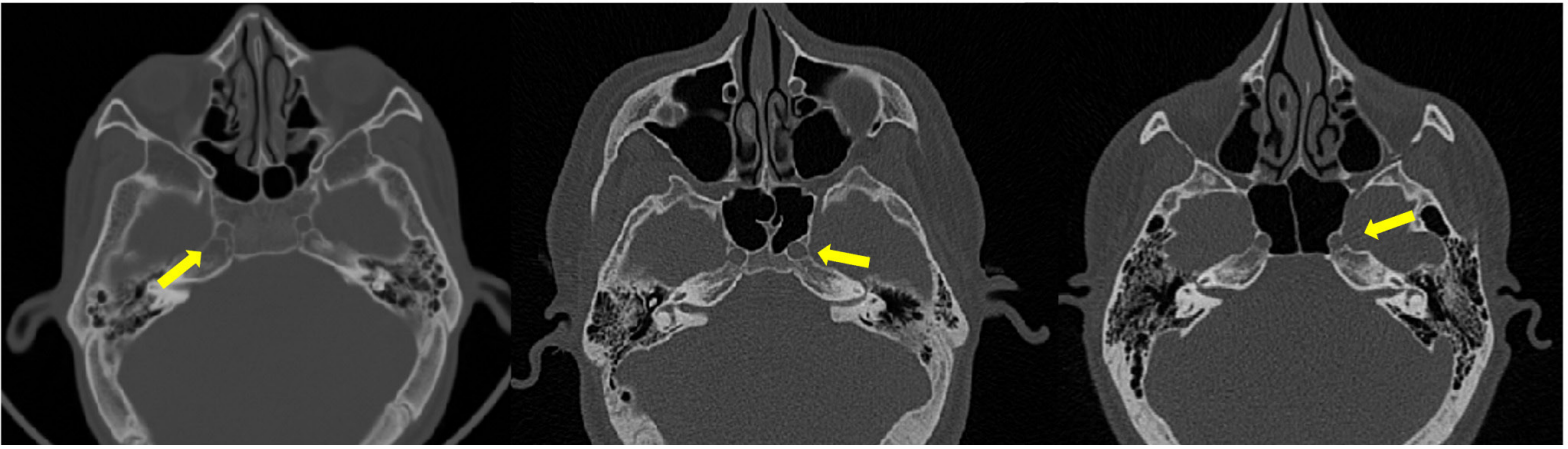
Classification of ICA in axial plane. (1) ICA does not protrusion the wall of the SS, (2) < 50% of the ICA protrudes into the sinus (3) > 50% of the ICA protrudes into the sinus.

**Figure 4:**
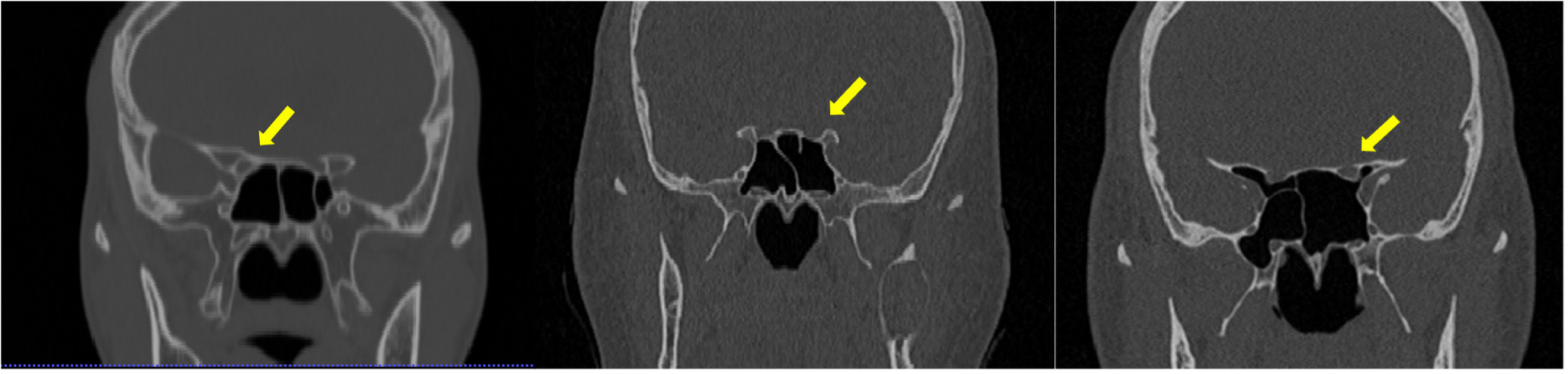
Classification of ON in coronal plane. (1) ON does not protrusion the wall of the SS, (2) < 50% of the ON protrudes into the sinus (3) > 50% of the ON protrudes into the sinus.

a) Pneumatization: regarding the type of extension, two anatomical points are: (1) the most anterior point of the anterior wall of the sella (AAS) and (2) the most posterior point of the posterior wall of the sella (PPS). Following Yamashita *et al*. classification (12), two tangents were drawn passing through the mentioned reference points and perpendicular to the Frankfurt horizontal plane (13). The first line represented the anterior limit of the pituitary fossa, while the –second representing the posterior limit of the pituitary fossa. The type of extension was classified as pneumatized, conchal, presellar, sellar, or postsellar (Fig. 1).

b) Extension: based on pneumatization in clivus classified as subdorsal, dorsal, occipital, and combined (dorsal and occipital) types based on posterior wall, floor of sellae, and vidian canal.

c) Septation: based on the number of intrasinusal septa present, quantified in relation to the presence of complete and incomplete septa.

d) Lobulation: classified according to the presence of intra-sinus lobules as bilobulated or multilobulated.

e) ICA and ON evaluation: assessed in the axial and coronal view, with results being classified as 0, 1, 2, or 3 based on the level or absence of protrusion (Figs. 2,3,4).

-Study error and statistical methods 

Two examiners were calibrated to establish uniform criteria for imaging evaluation. Cohen’s Kappa statistic was applied, obtaining an inter-rater agreement value higher than 0.80. Qualitative data were analyzed using Chi-square tests and frequency, represented in percentages (%). The level of statistical significance was set at *P* <0.05. The analyses were performed by an investigator (PGBS) using SPSS version 20.0 (IBM Corporation, Armonk, NY, USA), with a 95% confidence level.

## Results

A total of 320 patients were assessed using CT, with a mean age of 43.13 ± 17.39 years. The study population comprised 174 female and 146 male patients. No significant statistical difference in prevalence of pneumatization patterns between male and female subjects was observed.

The most prevalent classification type of pneumatization was the post sellar variation (60.0%), followed by the sellar (25.3%) and pre sellar (14.4%) variations ( Table 2). In relation to the extension, the most prevalent variation was the subdorsal (72.3%), followed by the occipital (13.9%) and dorsal (7.7%), with the combined type being the least prevalent (Table 2).

Septations either present as complete septa that extend from the anterior to the posterior wall dividing the sinus into different antra or as incomplete accessory septa. The majority (90.9%) of the patients presented with at least one main complete intersphenoidal septum, while 6.9% had both a complete intersphenoidal septum and at least two incomplete septa. The most prevalent was the presence of both types of septations being complete and incomplete in the same patient ( Table 3). Only one patient had no septa.

Protrusion of the ICA into the SS right and left sides was identified in 122 (38.1%) and 131 (40.9%) patients, respectively. Dehiscence of the bony sphenoidal wall of the right and left internal carotid artery occurred in 121 (37.8%) and 119 (37.2%) patients, respectively. From among the total participants, 161 (53.4%) and 199 (59.4%) exhibited ON protrusion into the right and left SSes, respectively ( Table 4).

Protrusion of the ICA into the SS right side was identified in 122 (38.1%) patients; the SS left side in 131 (40.9%). The dehiscence of the bony sphenoidal wall of the right internal carotid artery occurred in 121 (37.8%) patients and left side in 119 (37.2%). From the total participants, 161 (53.4%) cases had the optic nerve protrusion into the right sphenoid sinus and 199 (59.4%) in left side ( Table 4).

## Discussion

This study offers insights into the anatomic aspects of the SS. The examination of anatomic variations and pneumatization of the paranasal sinuses is significant in diagnosing and understanding sinus pathologies. The SS pneumatization may be genetic or environmentally influenced, resulting in great anatomical diversity (5). It is important to understand the internal anatomy of the SS due to its unique localization at the center of the skull and high anatomical variation. The SS is crucial to transsphenoidal endoscopic surgery as it facilitates access to the pituitary gland. During trans-sphenoidal surgery, surgeons should be aware of anatomical variances to prevent iatrogenic lesions including injury to the ICA and other critical structures proximal to the sinus at the base of the skull (2).

In the present study, in both males (59.2 %) and females (61.0 %), and across all age groups, postsellar type SS were more commonly detected. Sellar type was detected in the second order of both groups, while conchal type was rarely detected. The sphenoid region is considered by surgeons to be the most challenging area of the face due to its enclosure within the sphenoid bone and its proximity to numerous vital neural and vascular structures (9). These sinuses were initially classified into conchal, presellar, and sellar types (14), a widely accepted classification that served as a predictive tool for the surgical corridor in transsphenoidal surgeries. Modifications to the traditional system have since been introduced, focusing on the posterior extent of pneumatization and the ease of accessibility to the sellar floor during endoscopic surgeries (3).

The sphenoid may also be characterized by its extent of pneumatization. (15), describe the extent of its inferior–posterior pneumatization concerning the sela turcica using a modification of the classification proposed by Hammer and Radberg, 1962 (14). Two Asian cadaveric studies yielded similar results, with the sellar type being the most prevalent at 55%, and the least common being the presellar type at 17% (16). Another study involving 100 Korean cadaveric heads revealed that the sellar type was predominant at 90%, followed by the presellar type at 9% and the conchal type at 1% (17). The data obtained in the current study can prevent complications rhinologists may face during endoscopic surgical procedures. The conchal non-pneumatized sphenoid was always contraindicated for trans-sphenoidal approach to the sella (18). Therefore, the small percentage for conchal type pneumatization is considered to be better for our patients.

To analyze the anterior region of the skull base, methods such as cadaveric studies and plain radiographs have been replaced by CT assessments. Imaging methods that apply lower radiation than the conventional CT can serve as adequate digital models for the assessment of facial morphology, without compromising accuracy (19). CT of the paranasal sinuses is essential for a careful assessment of all structures in this region (4,5). A CT scan is one of the most accurate methods of obtaining medical images of the paranasal sinuses, as it provides a clear-cut representation of the osseous structures and identifies anatomical variations. Moreover, three-dimensional reconstructions may be created, providing additional insights into the complex anatomy of the SS (20), which is why we use it to evaluate the morphology and structures of the SS.

Differences in sinus type, size, presence of septa, and shape also exist among these diverse variations, are. Typically, the sinus presents as two antra that may be separated by a main intersphenoidal septum. However, it can also feature accessory septa or complete septa, either in the right or left cavity, extending from the anterior to posterior wall (21). Because the presence of septa can result in morphological variations of the sinus, recognizing its presence may help accurately identify the ICA, thereby reducing the risk of injury to it (22). The previously reported prevalence of the inter‑sphenoid septum insertion into ICA bony covering ranges from 4.9% to 89% (23,24,25) while in our study, the prevalence is 98.4%. During endoscopic surgery, the septum must be removed to expose the sella floor, thereby creating an adequate surgical corridor (24). Extreme caution is thus required while removing the septum in patients undergoing inter‑sphenoid septum insertion into the bony covering of ICA because an avulsion fracture of the septum may rupture the vessel, causing an intraoperative emergency. We found that the majority of the patients presented with at least one main complete intersphenoidal septum. The prevalence of accessory septations in different ethnic groups, which has been extensively investigated, reportedly ranges between 42% and 80% (1). This variant is influenced by ethnic variability (20,25-27).

The current study demonstrates that the sinus type is significantly associated with the frequency of the adjacent vital structure’s protrusions into the sinus. The relationship between the SS type and the protrusion of ICA and ON into the sinus has been assessed previously (4,28). A higher incidence of anatomical variations in the SS can pose an increased risk of injury to important neurovascular and glandular structures. The extensive hyper pneumatization of the SS, coupled with the consecutive pneumatization of the ethmoid sinus, may result in injury to the ON and protrusion of the internal ICA into the sinuses, potentially complicating surgical interventions (29). Numerous and frequent SS anatomical variants exist, which need to be comprehensively understood for successful surgical treatments and appropriate radiological interpretations. This in-depth analysis underscores the significance of categorizing these variances, explores their clinical ramifications, and reveals the demand for cutting-edge imaging methods and management approaches to improve patient outcomes. Our comprehension of these variances will be further increased by ongoing research and improvements in imaging methods, ensuring better patient care and surgical results. The prevalence of SS variants in various populations has to be further investigated. Studies focusing on the genetic and environmental factors influencing these differences may also offer insightful information about how they developed and possible connections to disorders associated with the sinuses.

Some limitations of this study need to be considered. Our sample consisted of Brazilian subjects only; hence, these findings need to be compared to other populations as well. The study included 320 tomographies from imaging centers located in northeastern Brazil (Salvador, Bahia; Belém, Pará; and Fortaleza, Ceará). The study locations were chosen because they are recognized reference centers for imaging. The detailed shape classification was based on a subjective grading. Apart from the number of septa, the relationships of these septa with neighboring structures is crucial in terms of surgical approaches. Information regarding the number of septa originating from or adhering to these structures is lacking, as is information about the group over the age of 50, that is, the elderly group. The aim was to report that the SS can present well defined and amorphous shapes. Therefore, the present findings should be interpreted with caution before any generalization is applied.

## Conclusions

In conclusion, sex and age play significant roles in the anatomical variations of the SS. The classification of neurovascular structures regarding their spatial association with the SS revealed no influence of sex and age. These findings highlight an important correlation between the anatomical variants of the SS and sex-specific as well as age-stratification data in a Brazilian ethnic group. Further studies are necessary to clarify the distribution of anatomical variability within the broader context of the general population.

## Figures and Tables

**Table 1:** Parameter definitions for SS evaluation.

Parameter	Classification	Definition
Pneumatization	Conchal	Slightly pneumatized, small sinus, unrelated to the sella turcica.
	Presellar	Pneumatization does not extend beyond the anterior limit of the sella.
	Sellar	Pneumatization does not extend beyond the posterior limit of the sella.
	Post-sellar	Pneumatization crosses the posterior limit of the sella.
Extension	Dorsal	Pneumatization extends superiorly into the dorsum sella.
	Subdorsal	Pneumatization doesn't extend below the vidian canal or into the dorsum sella.
	Occipital	Pneumatization extends inferiorly past the vidian canal.
	Combined	Sinus presents with both dorsal and occipital characteristics.
Septation	Complete	Septa which extend from one wall of the sinus to the next.
	Incomplete	Septa which dont extend from one wall to the next.
Lobulation	Unilobular	Absence of intersinusal septa
	Bilobular	Presence of one intersinusal septa
	Multilobular	Presence of multiple complete intersinusal septa
Internal Carotid (ICA) and Optic Nerve (ON)	Separeted	ICA/ON passes with significant distance to the sinus (0)
	Dehiscence	ICA/ON touches the sinus without protrusion (1)
	Protrusion <50%	Less than 50% of the canal diameter of the ICA/ON protrudes into the sinus (2)
	Protrusion > 50%	More than 50% of the canal diameter of the ICA/ON protrudes into the sinus (3)

**Table 2:** Sphenoid sinus type, symmetry, lobulation; and septation values in different age groups.

		Sex			18-25 years			26-49 years	
	Total	Men	Women	p-value	Men	Women	p-value	Men	Women	p-value
Classification										
Conchal	1 (0.3%)	1 (0.6%)	0 (0.0%)	0.734	1 (1.8%)	0 (0.0%)	0.579	0 (0.0%)	0 (0.0%)	0.896
Presellar	46 (14.4%)	27 (15.5%)	19 (13.0%)		12 (21.1%)	10 (15.4%)		15 (12.8%)	9 (11.1%)	
Sellar	81 (25.3%)	43 (24.7%)	38 (26.0%)		14 (24.6%)	19 (29.2%)		29 (24.8%)	19 (23.5%)	
Postsellar	192 (60.0%)	103 (59.2%)	89 (61.0%)		30 (52.6%)	36 (55.4%)		73 (62.4%)	53 (65.4%)	
Extension										
Dorsal	25 (7.8%)	7 (4.0%)	18 (12.3%)	0.028	4 (7.0%)	7 (10.8%)	0.464	3 (2.6%)	11 (13.6%)	0.009
Subdorsal	232 (72.5%)	129 (74.1%)	103 (70.5%)		46 (80.7%)	47 (72.3%)		83 (70.9%)	56 (69.1%)	
Occipital	45 (14.1%)	29 (16.7%)	16 (11.0%)		7 (12.3%)	9 (13.8%)		22 (18.8%)	7 (8.6%)	
Combined	18 (5.6%)	9 (5.2%)	9 (6.2%)		0 (0.0%)	2 (3.1%)		9 (7.7%)	7 (8.6%)	
Lobulation										
Unilobular	4 (1.3%)	2 (1.1%)	2 (1.4%)	0.001	1 (1.8%)	1 (1.5%)	0.084	1 (0.9%)	1 (1.2%)	0.008
Bilobular	295 (92.2%)	169 (97.1%)	126 (86.3%)		55 (96.5%)	56 (86.2%)		114 (97.4%)	70 (86.4%)	
Multilobular	21 (6.6%)	3 (1.7%)	18 (12.3%)		1 (1.8%)	8 (12.3%)		2 (1.7%)	10 (12.3%)	

*p<0,05, Fischer’s exact test or Pearson’s Chi squared test (n, %)

**Table 3:** Cases distribution according to sex and sinus septation.

		Sex			18-25 years			26-49 years		
	Total	Men	Women	p-Value	Men	Women	p-Value	Men	Women	p-Value
Complete Septa										
0	5 (1.6%)	3 (1.7%)	2 (1.4%)	0.007	1 (1.8%)	1 (1.5%)	0.114	2 (1.7%)	1 (1.2%)	0.102
1	291 (90.9%)	166 (95.4%)	125 (85.6%)		55 (96.5%)	55 (84.6%)		111 (94.9%)	70 (86.4%)	
2	22 (6.9%)	5 (2.9%)	17 (11.6%)		1 (1.8%)	8 (12.3%)		4 (3.4%)	9 (11.1%)	
3	2 (0.6%)	0 (0.0%)	2 (1.4%)		0 (0.0%)	1 (1.5%)		0 (0.0%)	1 (1.2%)	
Incomplete Septa										
0	105 (32.8%)	51 (29.3%)	54 (37.0%)	0.051	13 (22.8%)	24 (36.9%)	0.140	38 (32.5%)	30 (37.0%)	0.248
1	115 (35.9%)	64 (36.8%)	51 (34.9%)		24 (42.1%)	24 (36.9%)		40 (34.2%)	27 (33.3%)	
2	91 (28.4%)	57 (32.8%)	34 (23.3%)		20 (35.1%)	15 (23.1%)		37 (31.6%)	19 (23.5%)	
3	9 (2.8%)	2 (1.1%)	7 (4.8%)		0 (0.0%)	2 (3.1%)		2 (1.7%)	5 (6.2%)	

*p<0,05, Fischer’s exact test or Pearson’s Chi squared test (n, %)

**Table 4:** Relationship between dehiscence and the extend of protrusion of internal carotid artery and optic nerve.

		Sex			18-25 years			26-49 years		
	Total	Men	Women	p-value	Men	Women	p-value	Men	Women	p-value
Right ICA										
Irrelevant	77 (24.1%)	51 (29.3%)	26 (17.8%)	0.056	22 (38.6%)	14 (21.5%)	0.198	29 (24.8%)	12 (14.8%)	0.236
Touch	121 (37.8%)	63 (36.2%)	58 (39.7%)		17 (29.8%)	24 (36.9%)		46 (39.3%)	34 (42.0%)	
Protrusion										
< 50%	112 (35.0%)	57 (32.8%)	55 (37.7%)		17 (29.8%)	24 (36.9%)		40 (34.2%)	31 (38.3%)	
>50%	10 (3.1%)	3 (1.7%)	7 (4.8%)		1 (1.8%)	3 (4.6%)		2 (1.7%)	4 (4.9%)	
Left ICA										
Irrelevant	70 (21.9%)	43 (24.7%)	27 (18.5%)	0.197	16 (28.1%)	16 (24.6%)	0.494	27 (23.1%)	11 (13.6%)	0.263
Touch	119 (37.2%)	69 (39.7%)	50 (34.2%)		24 (42.1%)	21 (32.3%)		45 (38.5%)	29 (35.8%)	
Protrusion < 50%	122 (38.1%)	58 (33.3%)	64 (43.8%)		16 (28.1%)	26 (40.0%)		42 (35.9%)	38 (46.9%)	
>50%	9 (2.8%)	4 (2.3%)	5 (3.4%)		1 (1.8%)	2 (3.1%)		3 (2.6%)	3 (3.7%)	
Right ON										
Irrelevant	5 (1.6%)	5 (2.9%)	0 (0.0%)	0.091	1 (1.8%)	0 (0.0%)	0.559	4 (3.4%)	0 (0.0%)	0.157
Touch	144 (45.0%)	71 (40.8%)	73 (50.0%)		25 (43.9%)	31 (47.7%)		46 (39.3%)	42 (51.9%)	
Protrusion										
< 50%	155 (48.4%)	88 (50.6%)	67 (45.9%)		26 (45.6%)	31 (47.7%)		62 (53.0%)	36 (44.4%)	
> 50%	16 (5.0%)	10 (5.7%)	6 (4.1%)		5 (8.8%)	3 (4.6%)		5 (4.3%)	3 (3.7%)	
Left ON										
Irrelevant	1 (0.3%)	1 (0.6%)	0 (0.0%)	0.088	0 (0.0%)	0 (0.0%)	0.495	1 (0.9%)	0 (0.0%)	0.157
Touch	120 (37.5%)	57 (32.8%)	63 (43.2%)		19 (33.3%)	28 (43.1%)		38 (32.5%)	35 (43.2%)	
< 50%	184 (57.5%)	110 (63.2%)	74 (50.7%)		35 (61.4%)	33 (50.8%)		75 (64.1%)	41 (50.6%)	
> 50%	15 (4.7%)	6 (3.4%)	9 (6.2%)		3 (5.3%)	4 (6.2%)		3 (2.6%)	5 (6.2%)	
Age_CAT										
18-25	122 (38.1%)	57 (32.8%)	65 (44.5%)	0.031	-	-	-	-	-	-
26-49	198 (61.9%)	117 (67.2%)	81 (55.5%)		-	-		-	-	

*p<0,05, Fischer’s exact test or Pearson’s Chi squared test (n, %)

## Data Availability

The datasets used and/or analyzed during the current study are available from the corresponding author.
